# Acknowledgment of Reviewers

**DOI:** 10.2188/jea.JE20180237

**Published:** 2018-12-05

The following scientists assisted the journal by reviewing manuscripts during the period November 1, 2017 to October 31, 2018.

We gratefully acknowledge their critical evaluation and assistance in selecting articles for publication.

**Table:** 

Abrahamowicz, Michal
Akiyama, Naomi
Almeida-Pititto, Bianca de
Aoshima, Syuichi
Arima, Hisatomi
Asakura, Keiko
Asayama, Kei
Bae, Sanghyuk
Baik, Inkyung
Carreras, Giulia
Cederroth, Christopher
Chastin, Sebastian
Cheng, Yawan
Cho, Soo-Jeong
Choe, Byung-Ho
Choi, Il Ju
Chu, Anne
Doi, Suhail
Eguchi, Hisashi
Endoh, Kaori
Fowler, Helen
Fujieda, Shigeharu
Fujii, Ryosuke
Fujino, Yoshihisa
Fukuda, Yoshiharu
Fukuhara, Masayo
Furuta, Michiko
Ghiasvand, Reza
Goto, Atsushi
Guo, How-Ran
Hamano, Kumiko
Haruyama, Yasuo
Hashimoto, Hideki
Hata, Jun
Hayama-Terada, Mina
He, Yao
Higashiyama, Aya
Hikichi, Hiroyuki
Hilal, Saima
Hirata, Takumi
Hisamatsu, Takashi
Hishida, Asahi
Hojo, Masayuki
Hori, Megumi
Horikawa, Reiko
Hosono, Akihiro
Hosono, Satoyo
Ichikawa, Masao
Ikeda, Ai
Irie, Koichiro
Ishikawa, Shizukiyo
Ishikawa-Takata, Kazuko
Ishimaru, Miho
Isumi, Aya
Ito, Jun
Iwasaki, Masanori
Iwasaki, Motoki
Jee, Sun Ha
Jinnouchi, Hiroshige
Kachi, Yuko
Kakizaki, Masako
Kanamori, Satoru
Katanoda, Kota
Kato, Tsuguhiko
Katsuda, Nobuyuki
Kawano, Reo
Kikuchi, Hiroyuki
Kim, Hyeon Chang
Kitano, Naomi
Kobayashi, Minatsu
Kojima, Masayo
Kondo, Atsuo
Kondo, Shinsuke
Koriyama, Chihaya
Koyama, Shihoko
Kubota, Yasuhiko
Kumar, Santhosh
Kurotani, Kayo
Kuwahara, Keisuke
Lee, Jong-Tae
Leventhal, John
Lima, Marina
Liu, Xiaoqiu
Lobo, Elena
Lopes, Ana Isabel
Lugo, Alessandra
Lupo, Philip
Marklund, Matti
Martinez-Sanchez, Jose Maria
Matsuda, Tomohiro
Matsue, Yuya
Matsui, Hiroki
Matsuki, Hideaki
Matsuo, Keitaro
Matsushita, Sachio
Matsushita, Yumi
Matsuyama, Yusuke
Matsuzaka, Masashi
Metoki, Hirohito
Michikawa, Takehiro
Minami, Ushio
Minicozzi, Pamela
Miyagawa, Naoko
Momma, Haruki
Morisaki, Naho
Morishima, Toshitaka
Muhsen, Khitam
Murakami, Keiko
Muraki, Isao
Murata, Atsuhiko
Murata, Chiyoe
Murayama, Hiroshi
Murayama, Nobuko
Nagai, Masato
Nagamine, Yuiko
Nagata, Chisato
Nakagawa, Hiroko
Nakamura, Mitsuhiro
Nakamura, Yosikazu
Nakata, Yoshio
Nakayama, Masaharu
Nakayama, Takeo
Nanjo, Kishio
Ninomiya, Toshiharu
Nishi, Nobuo
Nishikitani, Mariko
Nishino, Yoshikazu
Noma, Hisashi
Noto, Hiroshi
Obara, Taku
Oh, Changmo
Oh, Jin-Kyoung
Ohara, Tomoyuki
Ohkusa, Yasushi
Ohnishi, Hirofumi
Ohsawa, Masaki
Okamura, Tomonori
Okumura, Yasuyuki
Ono, Sachiko
Otsuka, Rei
Ozasa, Kotaro
Oze, Isao
Park, Boyoung
Rajindrajith, Shaman
Rodo, Xavier
Rosen, John
Runyan, Desmond
Ryo, Okubo
Sado, Mitsuhiro
Saika, Kumiko
Saito, Eiko
Saito, Isao
Saito, Junko
Saito, Shinji
Saito, Tami
Saito, Toshiyuki
Sakurai, Ryota
Sangthong, Rassamee
Sasai, Hiroyuki
Sata, Fumihiro
Sawada, Takayuki
Schimmel, Michael
Seino, Yutaka
Shaw, Benjamin
Shibata, Ai
Shim, Jung Yeon
Shimazaki, Yoshihiro
Shimizu, Yuji
Shimoya, Koichiro
Shiner, Brian
Shinkai, Shoji
Shinozaki, Tomohiro
Shioda, Kayoko
Shirai, Kokoro
Shivappa, Nitin
Shobugawa, Yugo
Sloan, Robert
Sobue, Tomotaka
Sohn, Cheongmin
Sone, Hirohito
Staff, RT
Sugiyama, Daisuke
Sugiyama, Hiromi
Sugiyama, Kemmyo
Sugiyama, Takehiro
Surkan, Pamela
Suzuki, Etsuji
Suzuki, Kohta
Suzuki, Koji
Suzuki, Satoru
Suzuki, Shunji
Suzumori, Nobuhiro
Tabuchi, Takahiro
Taguri, Masataka
Takachi, Ribeka
Takahara, Mitsuyoshi
Takahashi, Kunihiko
Takahashi, Yoshimitsu
Takegami, Misa
Takeuchi, Ayano
Takeuchi, Kenji
Takimoto, Hidemi
Tanabe, Naohito
Tanaka, Akihiro
Tanaka, Junko
Tanaka, Sachiko
Tanaka, Shiro
Tanigawa, Takeshi
Taniguchi, Yu
Tanihara, Shinichi
Taylor, Mark
Teo, Soo H
Teramoto, Norihiro
Thrasher, James
Tomata, Yasutake
Tourino, Luciana
Tsai, Elaine
Tsubota, Megumi
Tsuboya, Toru
Tsuchihashi, Yuuki
Tsuji, Taishi
Tsujimoto, Takehiko
Ueda, Kayo
Ueda, Yutaka
Uemura, Hirokazu
Ukawa, Shigekazu
Urayama, Kevin
Urushihara, Hisashi
Utada, Mai
Vo, Phuong
Wada, Hiroo
Wada, Keiko
Wakai, Kenji
Washio, Masakazu
Watanabe, Shuichiro
Wills, Thomas A.
Yamakawa, Michiyo
Yamamoto, Tatsuo
Yamazaki, Shin
Yokomichi, Hiroshi
Yokoyama, Yukari
Yoshida, Daigo
Yumura, Yasuhi
Zeng, Hongmei
Zhao, Wenhua
Zou, Yubao

